# Toward equity in cultivating a “*garden of mentors*:” An exploration of networking experiences in an implementation research training program

**DOI:** 10.1017/cts.2025.17

**Published:** 2025-02-11

**Authors:** Loni J. Parrish, Amanda Gilbert, Kate Hoppe, Gloria D. Coronado, Karen M. Emmons, Amy A. Eyler, Debra Haire-Joshu, Rebekah R. Jacob, Alison B. Hamilton, Shelly J. Kannuthurai, Ross C. Brownson

**Affiliations:** 1 University of Arizona Cancer Center, Tucson, Arizona, USA; 2 Prevention Research Center, Brown School at Washington University in St Louis, St Louis, MO, USA; 3 Department of Surgery, Division of Public Health Sciences and Alvin J Siteman Cancer Center Washington University School of Medicine, Washington University in St Louis, St Louis, MO, USA; 4 Harvard T.H Chan School of Public Health, Boston, MA, USA; 5 School of Public Health at Washington University in St. Louis, St. Louis, MO, USA; 6 Center for Diabetes Translation Research, Brown School at Washington University in St Louis, St Louis, MO, USA; 7 VA Center for the Study of Healthcare Implementation Innovation and Policy, VA Greater Los Angeles Healthcare System, Los Angeles, CA, USA; 8 Department of Psychiatry and Biobehavioral Sciences, University of California Los Angeles, Los Angeles, CA, USA

**Keywords:** Implementation science, mentoring, training program, networking, capacity-building program

## Abstract

**Introduction:**

The Institute for Implementation Science Scholars (IS-2) is a dissemination and implementation (D&I) science training and mentoring program. A key component of IS-2 is collaborating and networking. To build knowledge on effective networking and mentoring, this study sought to 1) conduct a social network analysis to determine whether underrepresented scholars have equivalent levels of connection and 2) gain insights into the differences in networking among racial/ethnic subgroups of scholars.

**Methods:**

Social network survey data were used to select participants based on number of collaborative connections (highest, lowest) and racial/ ethnic category (underrepresented, not underrepresented). Interviews were recorded, transcribed, and coded using an iterative process.

**Results:**

The sample consisted of eight highly networked scholars, eight less networked scholars, seven from underrepresented racial and ethnic groups, and nine from not underrepresented groups. Qualitative data showed a lack of connection, reluctance to network, and systematic issues including institutional biases as possible drivers of group differences. In addition, scholars provided suggestions on how to overcome barriers to networking and provided insights into how IS-2 has impacted their D&I research and knowledge.

**Conclusions:**

Underrepresented scholars have fewer network contacts than not underrepresented scholars in the IS-2 training program. It is imperative for leadership to be intentional with mentorship pairing, especially for underrepresented scholars. Future research might include interviews with program leaders to understand how network pairings are built to improve the mentorship experience.

## Introduction

Mentored training programs in dissemination and implementation (D&I) science create an environment where scholars can network and collaborate [1–5]. These capacity-building programs also aim to enhance scholars’ skills and knowledge in D&I competencies [2]. As more D&I training programs have developed, multiple gaps remain [3], including how to create equitable networking experiences and sustain relationships built during the training programs.

Given the magnitude of health disparities across multiple health conditions, there is a significant need to continue to build and expand D&I science training programs that focus on maximizing positive health behaviors and outcomes for all populations [7]. There are two main benefits of these types of capacity-building programs. First, research scholars receive the skills and knowledge they need to meet the growing demand of translating research to practice in ways that will ensure that their efforts address extant health equity issues [8]. Second, and perhaps most importantly, scholars have the opportunity to build their network in D&I science with peers, senior-level investigators, and community partners. These network connections enable scholars to increase their knowledge and access opportunities to use D&I science methods and strategies to address critical public health challenges [9].

Creating a robust research network and fostering collaborations are crucial for advancing D&I knowledge [1,10]. It is not realistic for scholars’ only source of mentorship to be the typical one-on-one mentor relationship, due to the high demands of researchers and the need to conduct team science in D&I research [10–12]. Research shows that transdisciplinary teams and social networks foster mentoring, training, scientific collaboration, and scientific productivity, all enhancing career development success [13]. Considering the benefits of a mentorship network, it is also important that training programs are more intentionally focused on equity. Promoting equity in a D&I training program focuses on creating a supportive environment that meets a variety of needs related to opportunities and advancement. Often in the workforce the needs of underrepresented groups are misread, which limits inclusivity and can further marginalize diverse individuals [14].

Using a mixed-methods approach that blended social network (quantitative) data with interview (qualitative) data, this study aimed to 1) use descriptive social network analysis to determine levels of connectivity across underrepresented scholars and 2) gain insights on how levels of connectivity shape scholars experience within the program.

## Methods

### Training description

Funded by the National Institutes of Health and the U.S. Veterans Administration, the Institute for Implementation Science Scholars (IS-2) program is a training program for early-stage and mid-career investigators interested in applying D&I methods and strategies to reduce the burden of chronic disease disparities. The program has trained 58 scholars in 3 cohorts, including 21 investigators (36%) from underrepresented racial and/or ethnic groups.

### Network data

Here, we briefly describe the IS-2 network survey and how we used its data to conduct sampling for qualitative interviews. Building on methods from a previous social network analysis [1], we evaluated the IS-2 network by examining the contact, mentor, and different collaboration connections across faculty and scholars. We conducted a social network survey annually, starting with each cohort before attending their first summer institute. The survey provided a complete list of names (roster), and for the last 12 months each respondent identified their frequency of contact (no contact, yearly, quarterly, and monthly), their mentoring relationship (mentored and/or mentored by) and types of collaboration they had engaged in within five areas: research, manuscript writing, co-teaching, co-presenting, and grant writing. New cohorts were subsequently added to the roster for each year. The first cohort (2020) participated in four years of social network survey data collection, while the last cohort (2022) participated in two at the time of this analysis.

We used average degree, or the average number of collaborations each person had within the network (any collaboration), as the main metric to describe highly networked members vs. those not highly networked (the top or bottom tertiles). We then compared these levels of connectivity across underrepresented (U) scholars and not underrepresented (NU) groups of scholars. The average collaboration for U scholars across 2020–2023 was 2.4 connections, compared to 4.3 for NU scholars. The current study sought to understand what might drive these differences among scholars.

### Qualitative data

We used purposeful sampling to interview a subset of IS-2 scholars to gain an understanding of their networks, specifically their experiences with mentoring and networking as IS-2 scholars. For the purposes of this study, a network can be internal, within the scholars’ organization or work unit or external, with others outside of the scholar’s organization (e.g., IS-2).

#### Interview guide development

We developed a semi-structured interview guide to understand the experiences of scholars in the IS-2 program. Building on a qualitative study from a previous training program [9], the interview guide addressed three key topics: 1) networking characteristics and value of networking; 2) challenges in networking; and 3) the contribution of IS-2 to the scholars’ networking. The interview guide was pilot tested for comprehension of the topics covered. The final interview guide is presented in additional file 1. Human Subjects approval was granted by the Washington University in St Louis IRB (#202212037, 202007186).

#### Participant selection

The team identified scholars through a widely used qualitive research technique, purposeful sampling [15]. We selected individuals from each of four groups based on the social network data, including underrepresented (U) and highly networked (HN), U and not highly networked (NN), not underrepresented (NU) and HN, NU, and NN. Once identified, we reached out to 17 scholars via email individually, including *n* = 9 from the 2020 cohort and *n* = 8 from the 2021 cohort, with an invitation to participate in a qualitative interview.

#### Qualitative interviews

Participants who agreed to participate were scheduled for a one-hour Zoom session. A research team member who is part of an underrepresented group interviewed all underrepresented scholars. Verbal consent to participate was obtained at the beginning of the Zoom session. Zoom interviews were recorded with permission and the audio from each interview transcribed verbatim and deidentified for analysis. Interviews were conducted between January 2023 and February 2023. Participants who completed the interview were offered a $25 gift card.

#### Codebook development

The authors developed the codebook, which was initially structured to align with each item of the interview questionnaire and based on an initial reading of five randomly selected transcripts. Further codebook development was done iteratively and through consensus-building meetings.

#### Coding and summarization process

Two authors completed all coding (AG and LP) with another serving as a tie breaker as needed (KH). Coding was conducted independently in Microsoft Word using deductive coding to assign codes to the data. The authors met to discuss the coding of each transcript to achieve consensus.

## Results

### Interview participant characteristics

We invited 17 scholars to participate in interviews. One scholar agreed to participate but was unavailable during the interview period. Therefore, 8 scholars from the 2020 cohort and 8 scholars from the 2021 cohort were interviewed. Among the 16 scholars, 7 were identified as underrepresented, and 8 were highly networked.

### IS-2 contributions to scholar mentoring networks

Scholars noted that the IS-2 training program contributed to their research, implementation science knowledge, career path, and networking experience. Specifically, scholars mentioned that IS-2 helped secure grant funding, publish papers, and receive proposal feedback. It is important to note that scholars stated that the implementation science knowledge they received through the training program expanded their network and skillset and allowed them to serve as implementation science consultants on projects.

*“I was able to get exposure in terms of how to write good implementation science grants… I was able to get funded for not only one but two NIH proposals, and I also used that training to write a third proposal that is about to be funded by a pharmaceutical company.” (HN, U)*

### Characteristics of mentor networks

Overall, participant satisfaction with their internal and external networks varied by representation and networked status. These variations were further elaborated in participant discussions of the value of and satisfaction with networks.

#### Value of and satisfaction with networks

When asked specifically about the value of networking internally within their organization and externally outside of their organization (including through IS-2), participants discussed several benefits across their research, career path, personal lives, and general network-building.

**Figure 1.**
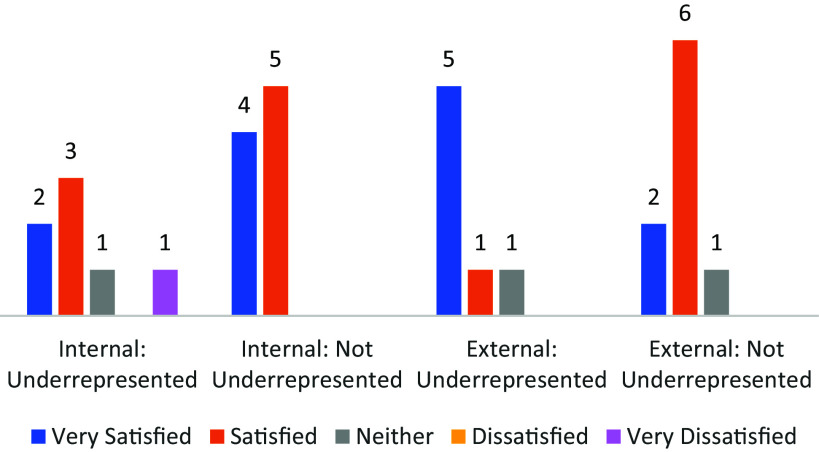
Satisfaction by network type and representation.

However, when broken down by representation, underrepresented scholars appeared less satisfied overall with their ***internal network*** than those who were not underrepresented (Fig. 1). Differences in overall responses about internal networks were similar to differences in satisfaction ratings. Underrepresented participants more often mentioned not having an internal mentor or not having one in their area of research and the importance of being at an institution that takes mentorship seriously.

*“…Here at the [institution], I’m the only one who’s doing [research topic] ….. So, if I need to build relationships, I either need to train folks in my department in these areas of research or look within the university…” (HN, U)*

Comparatively, participants who were not underrepresented more frequently mentioned long-term relationships with internal mentors spanning several years, with several participants indicating they were at the same institution where they did their PhD or postdoc.

*“So I would say the majority of my mentors are at my institution and I started at my institution as a postdoc fellow back four and a half years ago. And I’ve since had pretty much the same mentors from when I started my postdoc fellowship..” (HN, NU)*

There were differences in satisfaction with participants’ ***external networks by group.*** Underrepresented participants were more likely to be “very satisfied” with their external network compared to not underrepresented participants, who appeared more likely to be “satisfied” with their external network (including the IS-2 network) (Fig. 1).

These results were also mirrored in participant interview responses regarding their external networks. Both U and NU participants mentioned IS-2 about their external network. However, those who were highly networked mentioned IS-2 more frequently compared to participants who were not highly networked. Several highly networked participants referred to IS-2 mentorship as “invaluable.” Highly networked, underrepresented participants mentioned how instrumental IS-2 had been to providing an external mentor and supporting their work to write grant applications, secure grant funding, and other career-related endeavors. Those who were not underrepresented discussed IS-2 guidance and support within their area of interest and within implementation science.

*“So the mentor that I was paired with,,. mentorship has been invaluable. She was a big part in helping me with pushing through the process of writing a grant. My first really big grant on my own, it was one that was meant to kind of write it yourself” (HN, U).*

#### Networking advice

***Be intentional***. Intentionality includes purposefully choosing who to connect with and why and being aware of who is in the audience or at conferences. For not-highly networked respondents, intentionality also includes using mentorship pacts and agreements to facilitate communications and being intentional about the purpose and structure of mentorship relationships.

*“…to be intentional, to manage and grow your network and realize you’re the one in charge of that work. Nobody’s going to do it for you or tell you exactly how to do it or what you need.” (NN, U)*

***Seek diversity***. Seeking diversity was a theme for all groups except highly networked, underrepresented respondents. Seeking diversity involved identifying mentors and network connections to meet various needs. Acquiring knowledge, skills, and expertise requires connections to a diverse network.

*“…I think I’ve managed to cultivate a mentorship ground, if you will, or garden of mentors, who can help with different methods, who can help with leadership opportunities, who can help with scientific problems.” (NN, NU)*

***Know your needs***. All groups reported that knowing what you need to identify mentors and build an effective network is helpful.

*“…realizing the pros and cons of your network, or your strengths and weaknesses, and when you should use those individuals, or how to build a network to account for weaknesses that may be there.” (HN, NU)*

***Be present.*** Being present, engaged, and consistent were additional themes that emerged among highly networked respondents. Scholars reported that staying engaged with mentors and network connections, asking questions, and attending conferences, talks, and events was helpful.

*“I think staying consistent. I found that I would reach out to people and we’d have a great conversation and it would be fantastic. And then I never talked to them again, because everybody’s busy and everybody has their own other networks and other whatever. So if it’s someone that’s really important and pursuing them and keeping them on board, definitely staying engaged.” (HN, NU)*

***Put yourself out there***. The theme of not being afraid to put yourself out there was present in all groups except those who were not highly networked and not underrepresented.

*“I would say not being shy. And so one of my first instrumental mentors I met in the parking lot because I’d heard about her, I knew, I heard she was coming to the institution.” (NN, U)*

### Reasons for differences in networking among underrepresented scholars

When exploring scholar responses on why they thought the U scholars had fewer network contacts than NU scholars, three themes were identified: lack of connection, reluctance, and systemic issues.

#### Lack of connection

All groups mentioned that lack of connection could be another reason U scholars are less networked. It could be due to scholars from underrepresented groups not having senior mentors to connect them to others within the field. In addition, a common comment among scholars was that mentees connect better with people who have similar identities and share similar experiences and research areas.

*“I think we inherently seek similarness in people we work with in some way. It’s not only about race and gender, but that probably explains at least to some degree the differences in the size of the network or underrepresented racial ethnic groups. That’s what I would say.” (HN, NU).*

#### Reluctance

It is noteworthy that a common theme raised by U scholars is that this disparity in networking could be due to reluctance to reach out or lack of trust.

*“..as a person of color there is, for lack of a better way to say it, there is this feeling that for you to succeed, you have to pull from within, you have to rely on your own talent and capabilities. That only you can make something out of yourself. That just to rely or call on other individual seems like a bother, that these folks have lives, they’re very busy.” (HN, U).*

#### Systemic issues

All groups noted that systemic issues, including racism and not having emotional bandwidth, could be impacting the networking experience of underrepresented scholars.

*“There are so many biases and so many issues that come into play. But I think we need to have more opportunities for training, more opportunities for scholars to get in the room, more opportunities because you just never know what could happen. And it doesn’t always have to be the leading scholar with 25 NIH grants and 99 publications. You really need to be reaching back to the ones that don’t have that because they need the training. They need the mentoring….” (NN, U).*

*“So sometimes I think underrepresented individuals have a much bigger cognitive burden on this maybe unrecognized service aspect, that I think just may suck the life out of them…But I’m like, “I don’t know how they do it.”…, I probably can say no easier because of the privileges I mentioned earlier, which will free up time right? I can be much more selfish to build my network…” (HN, NU)*

## Discussion

D&I science training programs have the opportunity to develop the next generation of scholars whose research can improve health equity. Scholars need to be diverse, well-connected, and have a robust mentor network [16]. This study found differences in actual networks, satisfaction with networks, and reasons for the lack of networks among U and NU scholars.

Our study revealed that U scholars exhibited lower satisfaction with their internal network at their home institution, while reporting greater satisfaction with their external network, compared to their NU individuals. This phenomenon might suggest that U scholars prioritize training programs like IS-2, possibly due to low satisfaction with their internal networks or more limited resources at their home institutions. Prior research has consistently emphasized cultivating high-quality network contacts over sheer quantity [17,18]. Consequently, the elevated levels of external network satisfaction among U scholars could be attributed to their experience of fostering such quality network contacts.

In response to inquiries posed to scholars regarding the disparities in network contacts between U and NU scholars, several prominent themes emerged from the responses of both U and NU scholars. These themes included the issues of “lack of connection” and “systemic factors.” Notably, the theme of “reluctance” surfaced exclusively within the narratives of U scholars. These findings mirror previous research [19], highlighting the challenges faced by underrepresented faculty members in establishing robust mentoring relationships. Such challenges often stem from feelings of disconnection and the confinement of relationships to superficial interactions, thereby hindering their ability to establish meaningful connections [20].

Moreover, a report by the Kellogg School of Management augments these findings. It highlights that individuals from underrepresented backgrounds, particularly regarding socioeconomic status, tend to resist reaching out to others due to apprehensions of potential unreceptiveness [21]. These observations collectively underscore the critical nature of addressing the aforementioned themes and challenges in fostering more equitable and inclusive network experiences for scholars, particularly those who are underrepresented. Furthermore, as elucidated by scholars, the disparities in network contacts might be attributed to the cognitive load and the often-overlooked contributions made by U scholars. This perspective aligns with prior research findings that shed light on the U scholars’ challenges in balancing their demanding research commitments with their institutional service obligations. This burdensome role placed upon U scholars is commonly called the “minority tax” [22,23].

It is important that training programs address the underlying issues in networking. Programs should build confidence and while fostering meaningful connections. Furthermore, addressing systemic issues in academia, including promoting diversity in hiring and retention, and providing diversity, equity, and inclusion training is important because it can diversify and improve the quality of networking experience [16]. When systemic issues are actively confronted and rectified, networking and collaborations become more conducive to professional and personal growth [24].

### Implications for improving networking experiences for scholars

For those developing and implementing D&I training programs, we identify several implications from our experience that may lead to more positive networking experiences for scholars. Creating structured opportunities for scholars to collaborate and network with peers and senior researchers is helpful [9]. It is essential to intentionally create potential collaboration experiences and pair mentors with mentees (e.g., pairing U scholars with mentors highly skilled at networking). To support continued networking and collaboration, training programs can foster ***continued interaction with alumni*** through gatherings at professional meetings or collaborations on projects and publications [25]. We have found it helpful to provide a ***repository of materials*** to share and access educational information, job postings, and other career advancement materials.

## Future directions

The lessons learned from these findings can be used to improve the IS-2 training program and other related programs. Further research might include interviews with program leaders and program mentors to understand better how network pairings are built and to improve the mentorship experience for everyone involved. Future evaluations should include larger sample sizes for each subgroup of interest and studies from other scholar training programs.

## Conclusions

Our findings show that U scholars had a different networking experience than NU scholars in the IS-2 training program. Although U scholars had fewer network contacts within IS-2 than NU scholars, U scholars may be more satisfied with their external network than NU scholars. The difference in network connections could be due to a lack of confidence, opportunities for building connections, systemic issues, and reluctance. To foster a positive network environment, leadership must be intentional with mentorship pairing, especially for U scholars.

## References

[1] Brownson RC , Jacob RR , Carothers BJ , et al. Building the next generation of researchers: mentored training in dissemination and implementation science. Acad Med J Assoc Am Med Coll. 2021;96(1):86–92. doi: 10.1097/ACM.0000000000003750. PMC7769184 32941251

[2] Padek M , Colditz G , Dobbins M , et al. Developing educational competencies for dissemination and implementation research training programs: an exploratory analysis using card sorts. Implement Sci. 2015;10(1):114. doi: 10.1186/s13012-015-0304-3. 26264453 PMC4534127

[3] Straus SE , Sales A , Wensing M , Michie S , Kent B , Foy R. Education and training for implementation science: our interest in manuscripts describing education and training materials. Implement Sci. 2015;10(1):136. doi: 10.1186/s13012-015-0326-x. 26416302 PMC4585996

[4] Chambers DA , Proctor EK , Brownson RC , Straus SE. Mapping training needs for dissemination and implementation research: lessons from a synthesis of existing D&I research training programs. Transl Behav Med. 2017;7(3):593–601. doi: 10.1007/s13142-016-0399-3. 27030472 PMC5645270

[5] Davis R , D’Lima D. Building capacity in dissemination and implementation science: a systematic review of the academic literature on teaching and training initiatives. Implement Sci. 2020;15(1):97. doi: 10.1186/s13012-020-01051-6. 33126909 PMC7597006

[6] Brownson RC , Kumanyika SK , Kreuter MW , Haire-Joshu D. Implementation science should give higher priority to health equity. Implement Sci. 2021;16(1):28. doi: 10.1186/s13012-021-01097-0. 33740999 PMC7977499

[7] by Brownson ERC , Colditz GA , Proctor EK , Chambers, by FD. Dissemination and implementation research in health: Translating science to practice. Third Edition, New to this Edition:, Third Edition, New to this Edition: Oxford University Press; 2023.

[8] Friedman DB , Escoffery C , Noblet SB , Agnone CM , Flicker KJ. Building capacity in implementation science for cancer prevention and control through a research network scholars program. J Cancer Educ Off J Am Assoc Cancer Educ. 2022;37(6):1957–1966. doi: 10.1007/s13187-021-02066-3. PMC8266406 34240329

[9] Jacob RR , Gacad A , Pfund C , et al. The, secret sauce, for a mentored training program: qualitative perspectives of trainees in implementation research for cancer control. BMC Med Educ. 2020;20(1):237. doi: 10.1186/s12909-020-02153-x. 32723326 PMC7385963

[10] Puljak L , Vari SG. Significance of research networking for enhancing collaboration and research productivity. Croat Med J. 2014;55(3):181–183. doi: 10.3325/cmj.2014.55.181. 24891275 PMC4049205

[11] Aarons GA , Reeder K , Miller CJ , Stadnick NA. Identifying strategies to promote team science in dissemination and implementation research. J Clin Transl Sci. 2019;4(3):180–187. doi: 10.1017/cts.2019.413. 32695486 PMC7348006

[12] McGuier EA , Kolko DJ , Stadnick NA , et al. Advancing research on teams and team effectiveness in implementation science: an application of the exploration, preparation, implementation, sustainment (EPIS) framework. Implement Res Pract. 2023;4:26334895231190855. doi: 10.1177/26334895231190855. 37790168 PMC10387676

[13] Wolff HG , Moser K. Effects of networking on career success: a longitudinal study. J Appl Psychol. 2009;94(1):196–206. doi: 10.1037/a0013350. 19186904

[14] Saqib Z , Khan M. Striving for inclusion of diverse employees: how important is the context? South Asian J Hum Resour Manag. 2023;10(1):107–129. doi: 10.1177/23220937221083813.

[15] Palinkas LA , Horwitz SM , Green CA , Wisdom JP , Duan N , Hoagwood K. Purposeful sampling for qualitative data collection and analysis in mixed method implementation research. Adm Policy Ment Health. 2015;42(5):533–544. doi: 10.1007/s10488-013-0528-y. 24193818 PMC4012002

[16] Deanna R , Merkle BG , Chun KP , et al. Community voices: the importance of diverse networks in academic mentoring. Nat Commun. 2022;13(1):1681. doi: 10.1038/s41467-022-28667-0. 35338138 PMC8956734

[17] Pollack JM , Rutherford MW , Seers A , Coy AE , Hanson S. Exploring entrepreneurs’ social network ties: quantity versus quality. J Bus Ventur Insights. 2016;6:28–35. doi: 10.1016/j.jbvi.2016.09.001.

[18] Black P. Council post: Networking in the new normal: Choose quality over quantity. Forbes. Accessed November 13, 2023. https://www.forbes.com/sites/forbescoachescouncil/2021/08/27/networking-in-the-new-normal-choose-quality-over-quantity/.

[19] Lewis V , Martina CA , McDermott MP , et al. Mentoring interventions for underrepresented scholars in biomedical and behavioral sciences: effects on quality of mentoring interactions and discussions. CBE Life Sci Educ. 2017;16(3):ar44. doi: 10.1187/cbe.16-07-0215. 28747354 PMC5589424

[20] Markle RS , Williams TM , Williams KS , deGravelles KH , Bagayoko D , Warner IM. Supporting historically underrepresented groups in STEM higher education: the promise of structured mentoring networks. Front Educ. 2022;7:Accessed November 13, 2023 https://www.frontiersin.org/articles/10.3389/feduc.2022.674669.

[21] Why are some people more reluctant to network than others? Accessed November 2, 2023. https://insight.kellogg.northwestern.edu/article/network-size-social-status.

[22] Williamson T , Goodwin CR , Ubel PA. Minority tax reform - avoiding overtaxing minorities when we need them most. N Engl J Med. 2021;384(20):1877–1879. doi: 10.1056/NEJMp2100179. 34014047

[23] Trejo J. The burden of service for faculty of color to achieve diversity and inclusion: the minority tax. Mol Biol Cell. 2020;31(25):2752–2754. doi: 10.1091/mbc.E20-08-0567. 33253072 PMC7851863

[24] Wyatt GE , Hamilton AB , Milburn N. Mentoring to dismantle structural racism. Am J Public Health. 2023;113(S2):S94–S97. doi: 10.2105/AJPH.2023.307343. 37339420 PMC10282851

[25] Milburn NG , Hamilton AB , Lopez S , Wyatt GE. Mentoring the next generation of behavioral health scientists to promote health equity. Am J Orthopsychiatry. 2019;89(3):369–377. doi: 10.1037/ort0000415. 31070422 PMC7577403

